# Using the ICF in Ireland

**DOI:** 10.1186/1471-2458-11-S4-S5

**Published:** 2011-05-31

**Authors:** Anne Good

**Affiliations:** 1 ICF FDRG - Task Group Ethics and Human Rights

## Abstract

This paper reflects on the use of ICF in Ireland, taking as a case study the experience of the first National Disability Survey (NDS). There were four clear effects in Ireland of using ICF as a framework for the NDS: a) that a broader range of people with disabilities was encompassed; b) that the environmental factors included from the ICF were comprehensive and policy relevant; c) that both barriers and facilitators were incorporated into the model; and d) that a focus on research ethics was encouraged. Some general conclusions regarding the benefits and limitations of ICF based on this experience are also drawn.

## Introduction

This paper provides some reflections on the use of the ICF in Ireland, taking as a case study the experience of the first Irish National Disability Survey (NDS) which was conducted in 2006 by the Central Statistics Office (CSO). Two reports were published on the NDS by the CSO, the first in 2008 [1] and the second in January 2010 [2]. In addition, a number of methodological papers were presented during the period 2003 - 2010, including at meetings of the United Nations’ Washington Group on Disability Statistics. Full and comprehensive evaluation of the NDS, and its use of the ICF as the conceptual framework, is under way but not yet completed, although an overview by the CSO was included in its two reports. This paper outlines the preliminary conclusions of the author, based on the pilot report for the NDS (2004) [3], the CSO reports (2008, 2010) [1,2] and previous methodological papers. Further work is planned.

## Social movements and the state

New social movements, such as the disability rights movement, create new political demands and states may respond by changing policy and service provision configurations. If such change is to be evidence based, it requires new research agendas, new statistics and new data collection exercises. All of these processes have occurred under the impact of the disability movement across Europe, as well as in many other regions. Not surprisingly, they also impacted in Ireland where changes were influenced by national developments and also by developments at the EU and UN.

## National disability policy and data in Ireland, 1993-2010

In Ireland the story begins in the early 1990s with the emergence of a strong disability rights movement. This movement, which linked in to those of other countries in Europe, North America and the Antipodes, was, and is, focussed around a social understanding of disability, with the aspiration of achieving equality and human rights for people with disabilities, including the ending of segregation and the promotion of independent living. Like other social movements, such as the women’s movement, the disability movement made new demands on the Irish State. Where the State responded to these new demands, new research agendas and data collection needs emerged for State bodies.

All of these concerns will be familiar to those from many other countries. However, a significant Irish initiative which may be somewhat unusual, and which had important implications for disability research and data collection, was the establishment of a Commission on the Status of People with Disabilities (modelled on an earlier Commission on the Status of Women) which reported to government in 1996. The work of the Commission was informed by the largest country-wide consultation exercise ever undertaken in Ireland, with people with disabilities, their families as well as other stakeholders. The recommendations of the Commission were accepted by the government and helped shape the change agenda for the next decade. The key new characteristics of policy regarding people with disabilities advocated by the Commission included the ‘social model’ understanding of disability, the mainstreaming of disability into all policy fields and a commitment to equality.

Developing and implementing such a transformative agenda created new research needs. The Commission report had included some recommendations which focussed on research and data collection. These research and data elements of the Commission’s report were strongly influenced by the United Nations’ Standard Rules on Equalisation of Opportunities for People with Disabilities (1993). Therefore, they included a recommendation that Ireland begin to conduct the type of regular National Disability Surveys which were already undertaken in many other developed countries. Furthermore, a new state agency, called the National Disability Authority, was created in 2000 and given a strong research remit. In its early years the NDA conducted a major review of the existing knowledge base in relation to disability in Ireland and found it to be inadequate for the new policy environment. The NDA also initiated a planning project for the first Irish National Disability Survey between 2001 and 2004. As part of this process a pilot study was commissioned by the NDA [3] which examined options for the national survey, including investigation of the (then new) ICF as a possible framework. The pilot report, which recommended use of the ICF in the full survey, was accepted, approved by government and became the basis for the survey itself, which was carried out as a post censal survey in 2006.

## Key question

So the key question addressed in this paper is ‘Has the ICF provided a useful framework and language for meeting the new data requirements?’ The author’s conclusions are proposed based on the most significant use of the ICF in Ireland to date, i.e. the National Disability Survey.

I would argue that there were four clear effects in Ireland of using the ICF as the framework for the NDS, and these allow us to draw preliminary conclusions regarding the benefits and limitations of the ICF. These effects are:

• that a broader range of people with disabilities was encompassed;

• that the environmental factors included from the ICF were comprehensive and policy relevant;

• that both barriers and facilitators were incorporated into the model;

• that a focus on research ethics was encouraged.

I will address each of these effects in turn before drawing some general conclusions regarding the ICF based on this experience.

### Range of disabilities included

It is a core understanding in the ICF model that disability is not a dichotomous concept but a human experience which exists on a spectrum. Where the category of ‘disabled person’ is distinguished from that of ‘non disabled person’ along that spectrum will vary in different data collection exercises. This has been the case in Ireland in the two principal efforts at national level to gather data, including prevalence data, on disability, these being the Census (2002, 2006) [4] and the National Disability Survey of 2006. Table 1 shows the difference between the census and the NDS in terms of overall prevalence of disability and in terms of the estimated numbers of people with the various types of disability.

**Table 1. Prevalence of disability:** 

Prevalence of disability	Census 2006 (Sampling frame for NDS)	Prevalence 8.1%
	NDS 2006 (Main sample False negatives and false positives)	Final prevalence 18.5%
**Estimated numbers of persons with a disability**	Census disability sample	325,800 persons with disability
	NDS general population sample (estimated)	423,300 persons with disability
	**Total (estimated)**	**749,100 persons with disability**

(CSO, 2008:14 – 15)

In Table 1 we see that when the NDS used the census as a sampling frame and included both a large ‘yes’ sample and a smaller ‘no’ sample, it identified a significant rate of false negatives and a smaller rate of false positives. Estimates which extrapolated those false negatives and false positives to the general population indicated a prevalence rate of 18.5% as compared with the 8.1% reported by the Census. Clearly, the NDS included a much larger range of people with disabilities.

Table 2 shows the differences in estimates for nine disability types between the census and the NDS. These include, for example, intellectual and learning difficulties (71,600 people in the total population, according to the Census, compared with 126,100 estimated from the NDS) and pain (152,800 people in the total population, according to the Census, compared with 348,500 estimated from the NDS).

**Table 2:** Disability type: differences Census and NDS

	Census	NDS
Seeing	50,600	108,900
Hearing	57,600	97,700
Speech	35,300	53,200
Mobility and dexterity	184,000	334,800
Remembering and concentrating	113,000	187,700
Intellectual and learning	71,600	126,100
Emotional and psychological and mental health	110,600	192,500
Pain	152,800	348,500
Breathing difficulties	71,500	162,100

(CSO, 2008:15)

There are a number of possible explanations for the different prevalence rates resulting from these two data collection exercises. These include the questions which were asked and the way in which the data was collected: in one case via a census form filled out by a head of household on behalf of all members of the household present on census night, in the other case by an interview conducted in person by a trained interviewer (whose training included disability awareness training), in most cases with the person with disability themselves and with a clear and stated focus on disability. Clearly a disability survey can explore more details and aspects of the experience of disability and was more successful in identifying people with certain types of disability, who were less likely to be included on the census form, such as older people with chronic pain or children with moderate learning disabilities. Furthermore, the NDS questionnaire was designed using the ICF as a framework and this also had an impact on these results. However, it is not yet possible, to identify exactly what impact the ICF had as compared with, for example, disability focus or interviewer effect. The two reports on the NDS released by the CSO provide some reflections on the question of reported and estimated prevalence rates but further work is needed (CSO, 2008, 2010: 21-25 and 341- 4) [1,2].

### Environmental factors relevant to policy

The NDS was conducted in the year following a major government initiative in relation to disability policy. Entitled the National Disability Strategy [5], the plan included new legislation and funding, along with a strong focus on mainstreaming disability policy across all government activities. To advance this mainstreaming agenda, six government departments were instructed to develop what were termed ‘sectoral plans’ for improving their performance in relation to citizens with disabilities. These departments were the following:

• Health and Children

• Social and Family Affairs

• Transport

• Environment, Heritage and Local Government

• Communications, Marine and Natural Resources

• Enterprise, Trade and Employment

(DJELR, 2005)

All six departments recognised the need for improved data collection in relation to disability. They played an active role in advising the CSO on the development of the final NDS questionnaires [6]. The findings of National Disability Survey are seen to be a major resource in implementing their sectoral plans and some (for example the Department of Social Protection, formerly known as Social and Family Affairs ) are currently engaged in more detailed analyses of the NDS data relevant to their work than was included in the two general reports by the CSO.

In such analyses, what are termed environmental factors in the ICF, are seen as key to tackling disablement and enhancing enablement, whether in employment, education, transport or other areas of State policy and provision. In the NDS, the following environmental factors were investigated in detail and offer a potentially rich source of data for planning and evaluation:

1. Aids and appliance

2. Care and help from other people

3. Attitudes

4. Transport

5. Built environment accessibility

6. Education

7. Work and training

8. Social participation

9. Sport and exercise

(CSO, 2006, 2008, 2010).

Furthermore, the NDS results can be analysed by the following variables:

• Age

• Gender

• Geographical location

• Type of disability

• Combinations of these

(CSO: 2006, 2008, 2010)

so that patterns and variations can be explored, e.g. to identify the differing experiences of men and women with disabilities, of those in rural and urban areas, of those with physical disabilities as compared with those with mental health difficulties.

The following are illustrative examples of policy relevant environmental factors explored in the NDS.

1. Care services:

• 10% of respondents reported unmet needs for care services

• The main reasons given for the unmet needs were:

○ no service available

○ unable to afford

○ did not know who to contact

2. Work:

• 37% of respondents stated they were interested in working

• Aids and supports were often reported to be lacking

• 45% said the main support they needed was flexible working arrangements

### Barriers and facilitators in key areas of life

The ICF model facilitates investigation of factors which act as barriers to people with disabilities in their daily activities and their participation in social and economic life (disabling factors). It also facilitates investigation of factors which act as facilitators in the same areas (enabling factors). In some cases, for example attitudes within society, factors can have complex effects as both barriers and facilitators. Detailed and nuanced analysis is, therefore, helpful.

Some examples include:

1. Education

• modifications required

2. Work and Training

• reasons for not working

3. Social Participation

• attitudes

4. Sports and Exercise

• general health

A cross cutting, and complex factor is that of social attitudes to disability.

Attitudes can be both barrier and facilitator and the NDS findings reflect this nuanced understanding:

• family, friends and health care professionals were seen as most enabling;

• 32% of respondents reported difficulties in at least one major life area due to attitudes of other people;

• large variation was found in relation to type of disability: 63% among people with mental health difficulties reported attitudinal barriers, compared to 19% among people with breathing problems.

### Ethical guidance

The consultation process undertaken during the NDS pilot 2002 – 2004 revealed a significant problem in relation to negative previous experiences of research among people with disabilities which was often attributed to poor research practice and lack of attention to research ethics. The ethical guidelines included in the ICF were a good starting point in resolving this problem. Further work was then undertaken such as the development of special ‘Guidelines for Interviewers’ during the pilot, which were then used in the field staff training for the full survey (these guidelines were then also adapted and used by the UN Washington Group on Disability Statistics during field testing of proposed census questions on disability, 2004-7).

The NDA subsequently continued and expanded this work on research ethics, including by producing and distributing NDA Ethical Guidelines for Disability Research [7].

## Conclusions

To summarise: the disability movement developed and championed the social model of disability which has had major impact on policy oriented research and data collection. During this period of major change, use of the ICF has helped national research bodies in Ireland meet the challenge of creating new evidence. Four significant areas of impact have been identified and require further investigation. These are inclusion of a larger proportion of the population in consideration of disability; detailed consideration of environmental factors; recognition of both barriers and facilitators in enabling/disabling processes; and attention to ethical concerns to improve research quality.

This experience leads me to the following conclusions at this stage in the evaluation process:

• Including people with disability at all stages of the research process is both vital and beneficial;

• International cooperation, such as that which has been created around the ICF, means learning and best practice can be shared;

• Further work is needed to build on what has already been achieved by the ICF. My own priorities would be in the areas of more developed ethical guidance and rigorous attention to the inclusion of people with disabilities as partners in revision processes;

• There is also much which needs to be done to improve awareness of the strengths and also the implications of this new approach to disability research. Perhaps the most obvious is the need to explain and convince with regard to the issue of multiple prevalence rates rather than a single prevalence rate for disability, which is still often sought.

I hope that sharing these reflections on the Irish experience of designing and conducting the first National Disability Survey based on the ICF, will prove helpful to those who are also engaged in this transformative reconceptualisation of disability for new policies at national and international levels.

## Competing interests

The authors declare that they have no competing interests.
